# Supplementary material for: Comprehensive Review on the Impact of Chemical Composition, Plasma Treatment, and Vacuum Ultraviolet (VUV) Irradiation on the Electrical Properties of Organosilicate Films
Source: Polymers (Basel). 2024 Aug 5;16(15):2230. doi: 10.3390/polym16152230 (PMC11314954; doi:10.3390/polym16152230)
Supplement: Supplementary file 1 [file polymers-16-02230-s001.zip › polymers-3106371-supplementary.pdf]

Supplementary Materials

S1. Confirmation of ODC Formation in OSG Films by Means of XPS and UV-induced Luminescence

Confirming the presence of oxygen-deficient centers (ODC) via XPS, as it was done in ref. [46], poses a challenging and contentious endeavor. Figure 3 in the paper shows the deconvolution of the Si 2p level in a low-*k* dielectric (ethylene-bridged PMO) and non-stoichiometric SiO_x enriched with silicon. The deconvoluted sub-stoichiometric peaks Si^{3+} and Si^{2+} in the case of SiO_x suboxide were interpreted as indicative of ODCs. However, these peaks also serve as natural indicators of low-*k* dielectrics (Figure 3b) and could be attributed to the presence of terminal ($–O_2Si(–C)_2$ and $O_3Si–C$ groups known as D and T groups, respectively) [46,47].

As an additional confirmation of the presence of Si–Si bonds in an organosilicate low-*k* dielectric with ethylene bridges, the authors also used the results of a photoluminescence study [98]. The presence of an intense band with an energy of ~2.7 eV, and a less pronounced band with an energy of ~3.6 eV was observed after excitation by VUV light with energies of 5.1, 6.11, and 7.7 eV. It was assumed that the photoluminescence band with an energy of 3.6 eV and the excitation with an energy of 6.1 eV are due to oxygen divacancy. Half of the Stokes shift of the photoluminescence band with an energy of 3.6 eV is (6.1–3.6)/2 = 1.25 eV. This value is close to the thermal ionization energy of the trap, W_t = 1.2 eV. This relationship might suggest that the photoluminescence band at 3.6 eV and the excitation band at 6.1 eV are due to oxygen divacancy in SiO_2.

This study employed VUV light excitation with energy levels surpassing the bond strength of $Si–CH_3$ in the examined films. Consequently, even if this peak is associated with ODC formation, it's plausible that they could have formed during the photoluminescence measurements and are not intrinsic properties of the studied low-*k* films. Unfortunately, elemental analysis of the films post-photoluminescence study was not conducted, leaving us without any information regarding potential modifications to the films after VUV light exposure for the photoluminescence excitation (PLE). Furthermore, a more recent photoluminescence study of PMO films featuring several different bridges [102], with UV light excitation below the threshold for Si–C bond stability, led to the conclusion that the peak at 2.7 eV is not associated with ODCs. Utilizing materials with different compositions (Table S1) allows us to conclude that the peaks at 3.7–3.9 eV exclusively appear in the films with a benzene bridge, while the peaks at 3.3 eV appear in the films with an ethylene bridge. All films contained methyl terminal groups, with characteristic peaks at 2.85–2.9 eV. All the films except 1,4-phenylene (1,4-BB) were deposited with porogen, and for this reason, the peak at 4.2–4.35 eV was assigned to the porogen residue (Table S1). This conclusion aligns with those of Marsik and Krishtab [131,146].

Table S1. General characteristics of used organosilicate glass (OSG) films and photoluminescence (PL) energy as a function of the carbon groups. For example, peaks at 3.78–3.9 eV are only observed in films with benzene bridges, so they can be attributed to the benzene bridge. A similar approach is used for other groups. The brackets show the low-intensity peaks, which appear mainly after hard bake (HB).

Sample Number	Sample ID	Terminal Group ($–CH_3$)	Bridging Group	Porogen	Characteristics PL Peaks (eV)			
1-1	BTMSE/MTMS=47/53	YES	Ethylene	YES	-	3.3	2.9	4.2
1-2	BTMSE/MTMS=25/75	YES	Ethylene	YES	-	3.3	2.9	4.2
2-1	1,3,5/1,3-BB	NO	Benzene	YES	3.9	-	(2.90)	(4.85)
2-2	1,4-BB	NO	Benzene	NO	3.68	-	(2.86)	-
2-3	1,4-BB-p	NO	Benzene	YES	3.78	-	(2.86)	4.2
3-1	MTMS	YES	NO	NO	-	-	2.85	(4.35)
3-2	MTMS-p	YES	NO	YES	-	-	2.78	4.35
Assignment of PL peaks					Benzene bridge	Ethylene bridge	$–CH_3$ terminal	$C–H_x$

S2. Stress-Induced Leakage Current (SILC) in Low-*k* Dielectric

Stress-induced leakage current (SILC) is normally used for characterization of the gate leakage current of a MOSFET used in semiconductor physics. The increase of the leakage current occurs due to defects created in the gate oxide during electrical stressing. The low-*k* films used for this purpose were fabricated by technology proposed by Urbanowicz et al. [148], and the films were porogen residue-free. Therefore, in this case, the leakage current is defined by the low-*k* matrix as shown by Vanstreels et al. [147], and it gave a chance to study defects generation in the low-*k* matrix. Chen Wu showed that SILC increases to a critical value before final hard breakdown and is mainly driven by the injected charges. It is negligibly influenced by temperature and voltage. The physical nature of these stress-induced defects is suggested to be caused by the degradation of the Si–O based skeleton in the low-*k* dielectric. An increase in the exposure time of a low-*k* dielectric in a strong electric field leads to a shift in the current–voltage (*I-V*) characteristics to the lower electric field region; that is, it is accompanied by an increase in the leakage current (see Figure S1) [174].

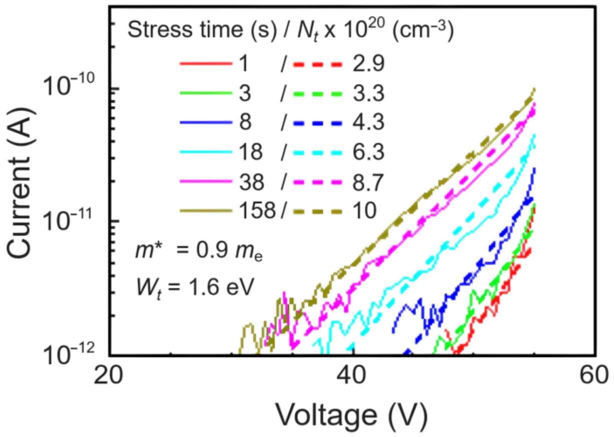

Figure S1. Experimental stress current versus stress time in the device after being subjected to high voltage stresses [174], as well as theoretical *I-V* (solid lines) simulated within the Nasyrov–Gritsenko charge transport model.

According to the N-G model of phonon-assisted electron tunneling between neighboring neutral traps, an increase in the trap concentration (N_t) is accompanied by an increase in leakage current. At $eF_s > kT$, the logarithm of the current is proportional to the electric field (Poole's law). The trap concentration is determined from the *I-V* slope on a semi-logarithmic scale.

It can be assumed that breakage of Si–O bonds, as suggested by Chen Wu, can further lead to the formation of Si–Si bonds (ODC) that then act as traps responsible for SILC in SiCOH low-*k* dielectric.

The σ-orbital of the Si–Si bond with two electrons coincides with the SiO_2 valence band top. The anti-bonding unfilled σ*-orbital of the Si–Si bond is localized near the conduction band bottom. It is assumed that during the stress current, hot electrons in collision with oxygen atoms contribute to the oxygen vacancy formation.

The agreement of the experimental and simulated within the N-G model *I-V* curves is observed at the thermal trap energy value W_t = 1.6 eV. This value is typical for SILC in the bulk of thermal SiO_2. The optical trap energy value in the thermal SiO_2 is equal to W_{opt} = 3.2 eV. This value is consistent with the trap values W = 3.4 eV and 3.6 eV with an accuracy of 15% obtained in ref. [172]. In this work, a tunnel trap filling mechanism is assumed. Earlier in ref. [175], it was shown that the optical trap energy coincides with the tunnel barrier for trap ionization in SiO_2. The correspondence of the trap parameters observed in SILC in the bulk of thermal SiO_2 with the trap parameters of the low-*k* dielectric is a strong argument in favor of the fact that there are traps associated with the Si–Si defect in the low-*k* dielectric.

S3. Simulation of experimentally measured *I-V* curves by Nasyrov–Gritsenko model

The major interest in this simulation was the possibility to see the difference between low-*k* films deposited by traditional PECVD technology with the following UV-assisted thermal curing and two films fabricated by the Urbanowicz approach and measured by different researchers (it is believed that these films are porogen residue-free) (Figure S2). In these papers, there is no current temperature

dependence, so the thermal and optical ionization trap energy values were taken to be 1.6 and 3.2 eV, respectively.

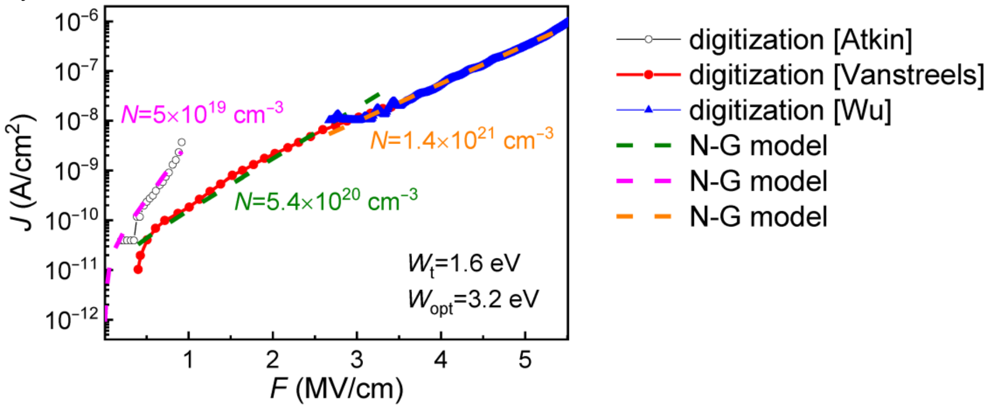

Figure S2. Experimental data from Atkin's paper [119] (black characters), from Vanstreels' paper [147] (red characters), from Wu's paper [174] (blue characters), and simulations with N-G model (dash lines) current–voltage (*I-V*) characteristics with different trap concentration values. For the N-G simulation, W_t = 1.6 eV, W_{opt} = 3.2 eV.

It is interesting that porogen residue films are extrapolated by the same line within N-G theory and give similar values of the traps concentration. PECVD low-*k* films that normally contain porogen residue give the traps concentration lower by an order of values. Further research of similar phenomena with low-*k* films with well-controllable composition is planned.
